# Trauma observation unit–based pathway for treatment of injured patients: A retrospective study

**DOI:** 10.5339/qmj.2026.24

**Published:** 2026-06-04

**Authors:** Ismail Mahmood, Bahaa Daoud, Mohammad Asim, Ismayil Chruveri, Muneer Poyil, Leni Udayan, Ahmad Ajaj, Zubaidah Alomar, Husham Abdelrahman, Ayman El-Menyar, Sandro Rizoli, Hassan Al-Thani

**Affiliations:** 1 Department of Surgery, Trauma Surgery, Hamad Medical Corporation, Doha, Qatar; 2 Department of Surgery, Qatar University, Doha, Qatar; 3 Department of Surgery, Trauma Surgery, Clinical Research, Hamad Medical Corporation, Doha, Qatar; 4 Jordan University of Science and Technology (Student), Irbid, Jordan; 5 Department of Internal Medicine, Weill Cornell Medicine – Qatar, Ar-Rayyan, Qatar

**Keywords:** Wounds and injuries, trauma, emergency service, observation units, patient safety, hospital readmission

## Abstract

**Background:**

Trauma remains a leading cause of hospital admissions and mortality worldwide. In the Emergency Department, Trauma Observation Units (TOUs) have emerged as an effective strategy to optimize care for patients who do not require inpatient admission.

**Objectives:**

To evaluate the outcome and safety of a TOU protocol on inpatient admission rates, length of stay, missed injuries, adverse outcomes, and 30-day emergency department (ED) readmissions among adult trauma patients.

**Methodology:**

We conducted a retrospective observational study of all adult patients admitted under the TOU protocol at Hamad Trauma Center, between 2023 and 2024. Data included demographics, trauma activation level, injury severity score (ISS), diagnostic test, missed injuries, length of stay, adverse outcomes, inpatient admission rates, and ED re-admission within 30 days. Descriptive statistics were used to summarize patient characteristics and outcomes, with data reported as proportions, means ± SD, or medians (range), as appropriate.

**Results:**

A total of 187 trauma patients were admitted to the TOU. The mean age was 37.6 ±14.9 years, with a predominance of males (154; 82.4%). The mean ISS was 5.5 ± 4.0. Frequent diagnoses included lacerations/soft tissue injuries (58; 31.0%), rib fractures (35; 18.7%), mild head injuries (29; 15.5%), and spinal transverse process fractures (19; 10.2%). The median duration of TOU stay was 10 hours (range 2–54). Repeated investigations were carried out in 36.4% of the total. Only one missed injury (1; 0.5%) occurred, without clinical consequence. Inpatient admission was required for 31 patients (16.6%), and 2 patients (1.1%) were readmitted within 30 days.

**Conclusion:**

TOU was safe and effective in selected trauma patients, with no adverse outcomes and minimal missed injuries. This model offers a patient-centered, resource-efficient alternative to routine admission. Future multicenter studies should validate outcomes, cost-effectiveness, and patient-reported measures.

## 1. INTRODUCTION

Trauma is the leading cause of hospitalization, disability, and death, imposing a substantial healthcare burden and financial strain.^1^ Notably, a considerable proportion of patients admitted to the emergency department (ED) are found not to have severe injuries enough to warrant inpatient admission.^2^ Nevertheless, these patients remain at risk of occult or evolving injuries, necessitating continued observation, additional diagnostics, and serial examinations to ensure safety and avoid missed diagnoses. In this context, TOU provides a practical care setting for patients who typically require admission for less than 24 hours.^3^ Current evidence indicates that ED-based Trauma Observation Units (TOUs) improve patient flow and efficiency. In a large scoping review, 41% of studies reported reductions in hospital length of stay, 37% evaluated lower admission or observation failure rates, and 35% demonstrated healthcare cost savings. Additionally, 26% of students directly compared observation unit care with inpatient admission, showing comparable or improved clinical outcomes without increased morbidity or mortality.^4^^,^^5^

Their integration into emergency and acute trauma workflows may alleviate the burden on trauma and emergency services, which are frequently challenged by high patient volumes.^6^

Accurate triage and close monitoring of trauma patients are essential, as certain injuries, particularly those with delayed clinical manifestations, may only become apparent through serial examinations and targeted investigations.^7^ The TOU provides a critical transitional phase of care, enabling comprehensive evaluation and safe observation of appropriately selected trauma patients. When applied correctly, observation-based management can reduce unnecessary inpatient hospitalization, with validated protocols demonstrating comparable or superior outcomes at lower cost across various emergency conditions.^8^^,^^9^

The Hamad Trauma Center serves as the only national Level I trauma referral center in Qatar and is among the busiest trauma facilities in the region, serving a population of approximately 2.8 million. According to the Qatar National Trauma Data Bank, nearly 2000 trauma activations are recorded annually.^10^ Despite this high trauma burden, evidence on the utilization and outcomes of TOUs within regional healthcare systems remains limited.

In response to high inpatient bed occupancy and the growing demand for trauma services, our level I trauma center implemented a Trauma Observation Protocol within the TOU. This protocol is designed to deliver efficient, safe, and high-quality care over short observation periods for carefully selected trauma patients.

Despite growing international evidence supporting the use of ED observation units, data specifically evaluating TOUs in trauma populations remain limited. There is a paucity of region-specific evidence from the Middle East regarding the safety, clinical outcomes, and resource utilization of trauma observation protocols. This knowledge gap limits the generalizability of current evidence and hinders informed implementation of trauma observation models in similar healthcare settings. Therefore, this study aims to evaluate the effectiveness and utility of a TOU protocol at the only Level I trauma center in Qatar, providing nationally relevant data and contributing to the international trauma observation literature.^11^

## 2. METHODOLOGY

A retrospective observational study included all patients admitted to Trauma Resuscitation Unit (TRU) under the trauma protocol at Hamad Trauma Center (HTC), Hamad General Hospital, Doha, Qatar, between March 2023 and May 2024. The HTC manages the care for approximately 2000 trauma activation patients per year at the only national Level I trauma center in the country. A consecutive sampling approach was used. All eligible patients admitted to the TOU under the trauma protocol during the study period were included. A total of 187 patients met the inclusion criteria and were analyzed.

### 2.1 Inclusion criteria

Patients were included if they met the following criteria:

- Age ≥14 years.
- Admission to the TOU under the trauma protocol.
- Blunt or penetrating traumatic injury suitable for short-term observation.
- Injury patterns including mild closed head injury, lacerations or soft tissue injuries, minor stab wounds, alcohol intoxication without significant associated injuries, simple rib fractures (≤3 ribs) without hemothorax or pneumothorax, or minor stable spinal fractures (e.g., isolated transverse or spinous process fractures managed conservatively).
- Pregnant trauma patients requiring short-term observation.
- Patients admitted at the discretion of the attending physician due to specific clinical or social considerations.

### 2.2 Exclusion criteria

Patients were excluded if they met any of the following criteria:

- Age <14 years.
- Abnormal vital signs at presentation.
- Multiple system traumatic injuries.
- Requirement for immediate inpatient admission or operative intervention.

Data were retrieved from the Qatar National Trauma Registry (QNTR) and electronic medical records. Data variables included patient demographics, mechanism of injury, injury characteristics, level of trauma activation, injury severity score (ISS), blood alcohol concentration, interventions, repeat X-rays, computed tomography (CT) scan, and laboratory investigations, missed injuries, length of stay in TOU, adverse outcomes (loss of vital signs, intubation, or death), inpatient admission rates, and ED re-admission rates. For discharged patients, clinical follow-up included documentation of any ED visits within 30 days of the initial presentation and identification of additional injuries diagnosed at those encounters.

### 2.3 Hamad TOU

The Hamad TOU is a six-bed monitored facility located within the ED at Hamad Trauma Center. It is designed to provide short-term observation, diagnostic evaluation, and stabilization for selected trauma patients whose admission, discharge, or transfer decisions cannot be accomplished within 3 hours but are anticipated within a 23-hour timeframe. The length of stay in the TOU is calculated from the time a trauma surgeon assigns the patient to the unit. Patients remain under the direct supervision of the trauma service (including the attending trauma surgeon, trauma specialist, trauma fellow, and resident) during observation, and those requiring care beyond the observation period are formally admitted under the appropriate service.^11^

Table 1 shows the TOU admission and management guidelines. Admission requires prior consultation with the trauma service, and patients may only be transferred to the TOU with approval. The trauma surgeon is responsible for completing the patient’s chart in the ED, overseeing all trauma-related concerns during observation, and ensuring adherence to specified management protocols. The trauma service must also document the initial consultation, issue standing orders for ongoing care, and prepare discharge documentation when appropriate. Patients requiring treatment beyond the 23-hour observation limit are to be formally admitted under the supervising service. Additionally, the TOU trauma surgeon is responsible for arranging follow-up clinic appointments when required.

### 2.4 Statistical analysis

Descriptive statistics were used to describe the characteristics of the trauma patients admitted to the observation unit. Categorical variables are presented as frequencies and percentages, while continuous variables are reported as mean ± standard deviation (SD) or median with range, as appropriate. All data analyses were performed using Statistical Package for the Social Sciences, version 21 (SPSS Inc., Chicago, IL).

The Medical Research Center (Institutional Review Board) approved the study protocol on August 7, 2024 (approval number: MRC-01-24-390), with a waiver of informed consent for the retrospective review of anonymized data from the QNTR database.

## 3. RESULTS

During the study period, a total of 187 patients were managed in the TOU, with a mean age of 37.6 years (±14.9), and a predominance of males (154; 82.4%). Table 2 presents demographics, injury characteristics, and outcomes of patients managed in the TOU. The most common mechanisms of injury were falls from height (58; 31.0%), motor vehicle collisions (36; 19.3%), and assault (25; 13.4%). The least frequent mechanisms included burns (2; 1.1%) and other causes (1; 0.5%). The most frequent diagnoses were lacerations, penetrating wounds, or soft tissue injuries (58; 31.0%), followed by rib fractures (35; 18.7%), mild head injuries (29; 15.5%), spinal transverse process fractures (19; 10.2%), and minor facial fractures (18; 9.6%). Other conditions included minor blunt abdominal trauma (12; 6.4%), alcohol intoxication (7; 3.7%), extremity fractures or dislocations (5; 2.7%), pelvic or pubic rami fractures (2; 1.1%), and burns (2; 1.1%).

Trauma activation levels (Table 3) were predominantly Level 2 (182; 97.3%), with only a few classified as Level 1 (4; 2.1%) or Level 3 (1; 0.5%). Ethanol was positive in 15 patients (8.3%). The mean ISS was 5.5 ± 4.0, reflecting the generally low to moderate severity of injuries.

The ISS is calculated by summing the squares of the three highest Abbreviated Injury Scale scores from different body regions. It ranges from 1 to 75, with scores of 1 to 8 indicating mild injury, 9 to 15 moderate, 16 to 24 serious, and ≥25 severe to critical.^12^

Repeat investigations, including laboratory tests (36.4%), X-rays (12.3%), and CT scans (7.0%). Only one missing injury (0.5%) was identified. The median length of stay in the TOU was 10 hours with a mean of 11.1 hours, ranging from 2 to 54 hours. However, five patients exceeded 23 hours, with four staying between 23 and 26 hours and one staying 54 hours.

Overall, 16.6% of patients were admitted to the ward; however, no adverse outcomes were reported during observation (Table 2).

## 4. DISCUSSION

The demographic profile of our cohort is consistent with patterns reported in other studies, where younger adults and male patients typically predominate due to greater exposure to high-risk environments and blunt trauma mechanisms.^6^^,^^13^^,^^14^ The mean ISS of 5.5 ± 4.0 is notably lower than the values reported in other TOU studies, which typically range from 9 to 14, as demonstrated by Muhamed et al.^6^ This difference likely reflects stricter patient selection criteria in our institution, favoring admission of lower-acuity trauma patients to the TOU, and may partly explain the lower complication and re-admission rates observed in our cohort. However, both are within the mild to moderate range.

The distribution of injury mechanisms in our cohort predominantly falls (31%), motor vehicle collisions (19.3%), and assaults (13.4%), which is consistent with previous literature, which also identifies falls and road-traffic injuries as the leading mechanisms in TOU populations.^6^^,^^14^^,^^15^ In contrast to other authors with a higher proportion of penetrating trauma, our cohort was largely composed of blunt injuries, which may limit direct comparability with centers serving different trauma populations.^8^

In terms of resource utilization, the frequency of repeated investigations in our cohort closely mirrors previously published data. Repeat X-rays were performed in 12.3% of our patients compared with 15.7% in prior work,^16^ and repeat CT imaging occurred in 7.0% versus 7.8% reported in the same study.^17^ Our mean length of stay of 11.1 hours aligns with other TOU findings from Muhamed et al. at 6.35 hours and Madsen et al. at 12.77 hours, which may reflect institutional constraints such as bed availability and discharge logistics.^6^^,^^16^

Our inpatient admission rate was 16.6% compared to Madsen et al.’s study, which was 11.5%.^16^ This difference may reflect a more cautious escalation strategy or differences in admission thresholds across institutions, rather than inferior TOU performance. Additionally, our re-admission rate of 1.1% compares favorably with the 3% to 15% range reported elsewhere as 4.6% and 15%,^16^^,^^18^ respectively. A prior 5-year retrospective observational cohort study conducted in New York, USA, involving 14,910 TOU encounters, reported that 15% of patients returned to the ED within 30 days.

Return visits were significantly associated with sex, ethnicity, race, insurance status, primary diagnosis, and disposition, but not with emergency severity index or length of stay.^18^ This finding reflects appropriate patient selection, ensuring that unnecessary admissions were minimized while avoiding premature discharges for patients requiring further care. Notably, the integration of TOU within trauma systems not only supports patient-centered care but also improves hospital flow and optimizes resource allocation.^13^^,^^15^ In high-volume urban trauma centers, where ED crowding and bed availability remain significant challenges, the TOU model offers a practical and scalable approach for managing trauma patients requiring short-term observation rather than immediate inpatient admission.^19^

Addressing both the similarities and differences relative to previously published studies, our findings are broadly consistent with the international literature on ED observation unit–based TOUs, which have been shown to safely manage selected trauma patients while reducing unnecessary hospital admissions. Prior studies by Muhamed et al.^6^ and Livingston et al.^14^ demonstrated favorable outcomes in narrowly defined trauma populations, such as blunt thoracic injuries and abdominal trauma with negative CT imaging, respectively. While these studies reported reduced hospital length of stay and low re-admission rates, their findings are limited to specific injury patterns. In contrast, our study evaluated a more heterogeneous trauma population, which may account for differences in outcome measures and limit direct comparison of effectiveness across studies.

Similarly, the mean and median lengths of stay observed in our cohort were comparable to those reported by Baugh et al.^20^ and others; however, prolonged observation in our study was primarily driven by system-level constraints, particularly inpatient bed availability, rather than clinical necessity. Furthermore, shorter stays in observation units contribute to improved patient flow within the ED, freeing up valuable inpatient beds for more critically ill patients.^21^ This highlights an important distinction between clinical efficiency and institutional operational factors, which are not uniformly captured across studies.

Taken together, our findings neither contradict nor unequivocally outperform existing evidence but instead suggest that TOU outcomes are highly context-dependent, influenced by patient selection criteria, injury severity, trauma case mix, and institutional resources. While the literature supports the safety and efficiency of TOUs in selected trauma populations, our results indicate that their performance metrics may vary when applied to broader trauma cohorts and different healthcare settings. This underscores the need for cautious interpretation of inter-institutional comparisons and highlights the importance of tailoring TOU implementation to local patient populations and system capacities.

Economic and system-level benefits of TOU have also been well-documented. A study by Perry et al.^22^ concluded that, in the setting of an academic medical center, the use of TOU resulted in more efficient resource utilization and lower healthcare expenditures, offering hospitals a valuable strategy to enhance operational efficiency and control costs. Within trauma care specifically, patients with minor thoracoabdominal injuries are managed in observation units versus inpatient wards. They reported comparable clinical outcomes, reinforcing the appropriateness of observation-based management for carefully selected trauma cases.^23^ These findings highlight the ability of observation units to maintain clinical vigilance while avoiding overtreatment.

The integration of physician-led triage with observation unit protocols has been shown to improve efficiency further, reducing the length of stay and enhancing patient flow and satisfaction.^24^ Collectively, these broader applications reinforce the role of TOU as a transitional care setting that bridges the gap between ED discharge and inpatient admission, ensuring appropriate resource utilization and minimizing overall healthcare costs.

### 4.1 Limitations

The strength of this study lies in its trauma-specific focus, addressing an area that has been less extensively studied compared to cardiac or general medical ED observation models. Nevertheless, several limitations must be acknowledged. First, the retrospective, single-center design may limit the generalizability of our findings. Second, although no adverse outcomes were reported, patient follow-up was limited to the duration of the observation period, thereby limiting the ability to capture delayed complications or long-term sequelae. Third, as most trauma cases were the result of blunt mechanisms, the generalizability of these findings to centers with a higher prevalence of penetrating injuries may be restricted. Fourth, given the retrospective and observational design, definitive conclusions regarding the comparative cost-effectiveness or safety of TOU care versus direct inpatient admission cannot be established. Finally, although patients had relatively mild injuries, standardized selection criteria for TOU admission remain necessary to ensure consistency across institutions.

### 4.2 Clinical recommendations and future implications

Future research should focus on comparative analyses between trauma patients managed in TOU versus traditional inpatient units to further evaluate differences in outcomes and to define best practices. Additionally, incorporating cost-effectiveness and patient-reported measures such as satisfaction, quality of life, and functional recovery will provide a more comprehensive understanding of the value of observation-based trauma care.

## 5. CONCLUSION

This study demonstrated that TOU provides safe and effective care for selected trauma patients. By facilitating timely clinical decision-making and reducing unnecessary hospitalizations, this model has the potential to enhance patient flow, optimize resource utilization, and maintain high standards of safety and satisfaction.

## ACKNOWLEDGEMENTS

We thank the trauma resuscitation unit at Hamad General Hospital, Doha, Qatar.

## FUNDING

The author(s) received no financial support for the research, authorship, and/or publication of this article.

## CONFLICT OF INTEREST

The author(s) declare that there is no conflict of interest.

## ETHICAL APPROVAL

The Medical Research Center (Institutional Review Board, Number: MRC-01-24-390) approved the study protocol, with a waiver of informed consent for the retrospective chart review of anonymous data.

## AUTHOR CONTRIBUTIONS

IM and ZA: Conceptualization; BD, IC, MP, LU, and IM: Methodology; MA: Formal analysis; ZA and IM: Data curation, writing—original draft preparation; HA, AA, AE, HA, and SR: Writing—review and editing. All authors have read and agreed to the published version of the manuscript.

## DATA AVAILABILITY STATEMENT

All data were presented in the manuscript and tables.

## DISCLOSURE OF AI USE

The authors declare that no artificial intelligence (AI) tools were used in the preparation of this manuscript.

## Figures and Tables

**Table 1.** Trauma Observation Unit (TOU) admission and management guidelines.

Section	Criteria/responsibilities
Responsibilities	Obtain Trauma Service consultation before TOU admission/transfer. Trauma Service provides consultation notes, standing orders, and discharge documentation. Patients requiring >23 hours of observation must be admitted to the supervising service.
Inclusion criteria	**Common injuries for observation:** – Simple rib fractures (≤3) without hemothorax, pneumothorax, or significant chest pain, in stable patients with no/minor comorbidities. – Alcohol intoxication without significant injuries. – Closed mild head injury with normal CT scan requiring observation. – Lacerations, minor stab wounds, and soft tissue injuries. – Minor stable spinal fractures (e.g., transverse process fractures) requiring simple measures (collar, pain management). – Physician’s discretion for specific needs (e.g., social issues).
Exclusion criteria	– Unstable vital signs (abnormal physiology). – Age <14 years. – Age >65 years with significant comorbidities. – Multi-system injuries.
Nursing staff	– Monitor vital signs. – Assess pain and neurological status. – Administer medications and assist with mobilization. – Document and escalate concerns.
Physiotherapy	– Assess mobility/function. – Initiate safe mobilization and breathing exercises.
Occupational therapy	– Evaluate activities of daily living. – Recommend assistive devices or home modifications before discharge.

**Table 2.** Demographics, injury characteristics, and outcome for patients placed in observation unit (*n* = 187).

Age (mean ± SD), years	37.6 ± 14.9
Males	154 (82.4%)
**Mechanism of injury**	
Fall from height	58 (31.0%)
Motor vehicle collision	36 (19.3%)
Assault	25 (13.4%)
Motorcycle crash	23 (12.3%)
Pedestrian	16 (8.6%)
Fall of a heavy object	12 (6.4%)
All-terrain vehicle	9 (4.8%)
Bicycle crash	5 (2.7%)
Burn	2 (1.1%)
Others	1 (0.5%)
**Diagnosis**	
Laceration/penetrating wound/soft tissue injury	58 (31.0%)
Rib fracture	35 (18.7%)
Mild head injury	29 (15.5%)
Spinal transverse process fracture	19 (10.2%)
Minor facial fracture	18 (9.6%)
Minor blunt abdominal trauma	12 (6.4%)
Alcohol intoxication	7 (3.7%)
Extremity fracture/dislocation	5 (2.7%)
Pubic rami fracture, pelvic/hip fracture	2 (1.1%)
Burn	2 (1.1%)
**Level of trauma activation**	
1	4 (2.1%)
2	182 (97.3%)
3	1 (0.5%)
Ethanol positive	15 (8.3%)
Injury Severity Score	5.5 ± 4.0
ED return/readmission within 30 days	2 (1.1%)
Repeat X-rays	23 (12.3%)
Repeat CT scan	13 (7.0%)
Repeat laboratory investigations	68 (36.4%)
Missed injuries	1 (0.5%)
Length of stay in observation, hours	10 (2–54)
Admission to the ward	31 (16.6%)
Adverse outcome	0 (0.0%)

**Table 3.** Hamad Trauma Center – Trauma Activation Criteria.

Level	Key activation criteria
**Level 1: Life-threatening**	Penetrating injury to neck/chest/abdomen/proximal limb Shock (SBP < 90 or absent pulses) GCS < 9 Severe bleeding Airway compromise or intubated at the scene Major vascular injury or proximal amputation Spinal cord injury Major impalement/unstable pelvis Significant trauma with pregnancy more than 20 weeks Physician discretion
**Level 2: Potentially life-threatening**	Penetrating extremity injury (distal to elbow/knee) Significant chest injury (flail chest/multiple ribs) Burns >20% or inhalation injury GCS 9–13 or open skull fracture Multiple long-bone fractures High-risk mechanism (ejection, pedestrian struck, fall > 4 m, high-speed Motor Vehicle Crash (MVC), occupant death, motorcycle crash
**Level 3: Non-life-threatening**	Stable patients without Level 1 or 2 criteria Single-system injuries
